# Genetic dissection of TrkB activated signalling pathways required for specific aspects of the taste system

**DOI:** 10.1186/1749-8104-9-21

**Published:** 2014-09-26

**Authors:** Juraj Koudelka, Jacqueline M Horn, Chinnavuth Vatanashevanopakorn, Liliana Minichiello

**Affiliations:** 1 Centre for Neuroregeneration, University of Edinburgh, EH16 4SB Edinburgh, UK; 2 Department of Pharmacology, University of Oxford, OX1 3QT Oxford, UK; 3 Department of Biochemistry, Faculty of Medicine Siriraj Hospital, Mahidol University, Bangkok 10700, Thailand

**Keywords:** Brain derived neurotrophic factor, Neurotrophin-4, TrkB signalling, Gustatory system, Survival, Taste buds, Taste receptors

## Abstract

**Background:**

Neurotrophin-4 (NT-4) and brain derived neurotrophic factor (BDNF) bind to the same receptor, Ntrk2/TrkB, but play distinct roles in the development of the rodent gustatory system. However, the mechanisms underlying these processes are lacking.

**Results:**

Here, we demonstrate, *in vivo*, that single or combined point mutations in major adaptor protein docking sites on TrkB receptor affect specific aspects of the mouse gustatory development, known to be dependent on BDNF or NT-4. In particular, mice with a mutation in the TrkB-SHC docking site had reduced gustatory neuron survival at both early and later stages of development, when survival is dependent on NT-4 and BDNF, respectively. In addition, lingual innervation and taste bud morphology, both BDNF-dependent functions, were altered in these mutants. In contrast, mutation of the TrkB-PLCγ docking site alone did not affect gustatory neuron survival. Moreover, innervation to the tongue was delayed in these mutants and taste receptor expression was altered.

**Conclusions:**

We have genetically dissected pathways activated downstream of the TrkB receptor that are required for specific aspects of the taste system controlled by the two neurotrophins NT-4 and BDNF. In addition, our results indicate that TrkB also regulate the expression of specific taste receptors by distinct signalling pathways. These results advance our knowledge of the biology of the taste system, one of the fundamental sensory systems crucial for an organism to relate to the environment.

## Background

It is well known that the rodent gustatory system develops under tight control of two neurotrophins, brain derived neurotrophic factor (BDNF) and neurotrophin-4 (NT-4), preferentially activating their high affinity receptor TrkB [1,2]. Each of these neurotrophins has been previously shown to regulate the survival of about half of the geniculate ganglion neurons, with additive losses observed in newborn mice lacking both BDNF and NT-4 [3,4]. Recently, it was also established that BDNF and NT-4 influence geniculate neuron survival at different developmental stages. Whereas NT-4 is necessary earlier in development, prior to target innervation, BDNF is mainly required to support differentiated geniculate neuron survival when they first innervate their target [5,6]. Furthermore, it has been shown that although both neurotrophins are involved in fibre branching from the base of the tongue to the epithelium, only BDNF is required for taste innervation and taste bud morphology [7]. These distinct functions of the two neurotrophins in the taste system development are mainly due to their different spatio-temporal expression patterns, since most of the defects arising in absence of BDNF in this system can be rescued by expressing NT-4 in the BDNF locus [1,2].

Since the gustatory system phenotype of *Bdnf*/*Nt4* double null mutants mirrors that of *Trkb* null mice, it has been suggested that BDNF and NT4 mediate their effects through TrkB [4,8-10]. Upon binding of either neurotrophin to TrkB, several well-characterized intracellular signalling pathways are activated as a result of the phosphorylation of tyrosine (Y) residues in the intracellular region of TrkB. In particular, phosphorylation of Y515 in the juxtamembrane domain, or Y816 in the carboxyl terminus, leads to the recruitment of the adaptor molecules Sh2-domain containing protein (SHC)/fibroblast growth factor receptor substrate 2 (FRS2) and phospholipase Cγ1 (PLCγ1), respectively, with subsequent downstream activation of their respective signalling pathways [11,12].

In order to dissect the contributions of the SHC and PLCγ docking sites of TrkB in taste system development, and at the same time to determine which of these sites mediate the effect of BDNF and NT-4 in this system, we have studied mouse lines in which Y515 or Y816 have been mutated either singly or in combination [13-15]. Interestingly, we report here that signalling through the TrkB-SHC docking site controls both early NT-4-dependent and the late BDNF-dependent gustatory neuron survival, since mice carrying a mutation in the TrkB-SHC docking site had reduced gustatory neuron survival at both early and later stages of development. The TrkB-SHC docking site also regulates geniculate neuronal innervation and taste bud morphology, both BDNF-dependent functions. In contrast, whereas the TrkB-PLCγ docking site is dispensable for gustatory neuron survival, it is required for the timing of their innervation of the tongue, which is BDNF-dependent, and for the appropriate expression of taste receptors (TRs). Therefore, these two major docking sites play unique and overlapping biological activities exerted by NT-4 and BDNF at different times during the development of the taste system.

## Results and discussion

### The TrkB-SHC docking site is necessary and sufficient to mediate TrkB-dependent geniculate neuron survival

**Figure 1:**
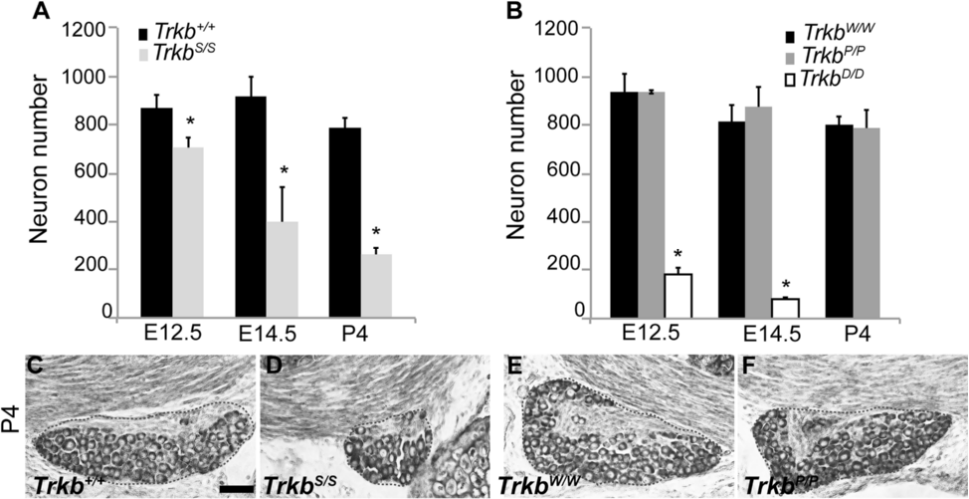
**Signalling activated through the TrkB-SHC docking site supports survival of geniculate neurons. (A)** Counts of geniculate ganglion neurons in E12.5, E14.5, and P4 *Trkb*^*S/S*^ animals and respective control littermates (*Trkb*^*+/+*^). Neuronal loss is detected already at E12.5 and increases at later stages (E12.5, *Trkb*^*S/S*^ 702 ± 47 (n = 3) vs. *Trkb*^*+/+*^ 869 ± 51 (n = 4), *P* <0.01; E14.5, *Trkb*^*S/S*^ 398 ± 142 (n = 3) vs. *Trkb*^*+/+*^ 915 ± 81 (n = 3), *P* <0.001; P4, *Trkb*^*S/S*^ 261 ± 32 (n = 2) vs. *Trkb*^*+/+*^ 789 ± 38 (n = 4), *P* <0.001). **(B)** Mutation at the PLCγ-docking site does not affect geniculate neuron survival at any stage analysed (E12.5, *Trkb*^*P/P*^ 934 ± 75 (n = 2) vs. *Trkb*^*W/W*^ 936 ± 62 (n = 4), *P* = 0.975; E14.5, *Trkb*^*P/P*^ 878 ± 77 (n = 6) vs. *Trkb*^*W/W*^ 815.5 ± 67 (n = 3), *P* = 0.279; P4, *Trkb*^*P/P*^ 790 ± 76 (n = 3) vs. *Trkb*^*W/W*^ 799 ± 37 (n = 3), *P* = 0.852). Survival analysis of geniculate neurons from *Trkb*^*D/D*^ showed substantial loss already at E12.5 (E12.5, *Trkb*^*D/D*^ 183 ± 27 (n = 3) vs. *Trkb*^*W/W*^ 936 ± 62 (n = 4), *P* <0.001; E14.5, *Trkb*^*D/D*^ 84 ± 5 (n = 3) vs. *Trkb*^*W/W*^ 815.5 ± 67 (n = 3), *P* <0.001). Values shown are mean ± s.d., and the *P* statistic from unpaired Student’s *t*-tests. **(C–F)** Representative images of Nissl-stained sections of geniculate ganglions from *Trkb*^*+/+*^_,_*Trkb*^*S/S*^, *Trkb*^*W/W*^, and *Trkb*^*P/P*^ mice at P4. Scale bars 50 μm.

Loss of *Trkb* greatly impairs the development of the gustatory system [8,10]. Deletion of *Bdnf* or *NT-4*, which acts primarily through TrkB receptors, affects distinct aspects of gustatory system development according to their different spatio-temporal expression patterns [2,5,6]. Therefore, we have studied mice carrying either single (TrkB^SHC^, TrkB^PLC^) or double (TrkB^D^) point mutations in two specific phosphorylation sites of TrkB [13-15] in order to determine how TrkB receptors influence the gustatory system development, and to examine the roles of these sites in mediating NT-4- and BDNF-dependent functions in the taste sensory system. We first determined which TrkB docking site is able to facilitate survival of geniculate neurons early or late in development. Geniculate ganglion neuron numbers were obtained at embryonic day 12.5 (E12.5), E14.5, and postnatal day 4 (P4) from the following homozygous mutant genotypes and respective controls, *Trkb*^*S/S*^ and *Trkb*^*+/+*^, *Trkb*^*P/P*^, *Trkb*^*D/D*^, and *Trkb*^*W/W*^ (see Methods). Neuronal counts at E12.5 revealed a significant loss (~20%) of neurons in *Trkb*^*S/S*^ mutants compared with wild-type littermates (Figure 1A). Cell loss continued during development and after birth when at P4 almost all TrkB-dependent geniculate neurons were lost (Figure 1A,C,D). In contrast, mutation of the TrkB-PLCγ adaptor site did not affect geniculate neuron survival during embryonic development or postnatally (Figure 1B,E,F). Analysis of the geniculate ganglion in animals carrying a mutation at both the SHC- and the PLCγ-sites (*Trkb*^*D/D*^) revealed them to be phenocopies of *Trkb* null mutants, showing 80% loss at E12.5 and 90% loss by E14.5 (Figure 1B). The latter corresponds to all of the TrkB-dependent geniculate neurons, since a small population of these neurons has previously been shown to be TrkB-independent [10].

These results indicate that signalling from the TrkB-SHC docking site largely regulates survival of taste neurons first by transducing NT-4 signalling, followed by the later onset BDNF signalling. Whilst signaling from the TrkB-PLCγ docking site is dispensable for geniculate neuron survival, this site nonetheless supports it, at least to a limited extent, since all TrkB-dependent taste neurons are lost in mice carrying both point mutations (*Trkb*^*D/D*^).

### Point mutation at the TrkB-PLCγ adaptor site delays taste bud innervation during embryonic development

**Figure 2:**
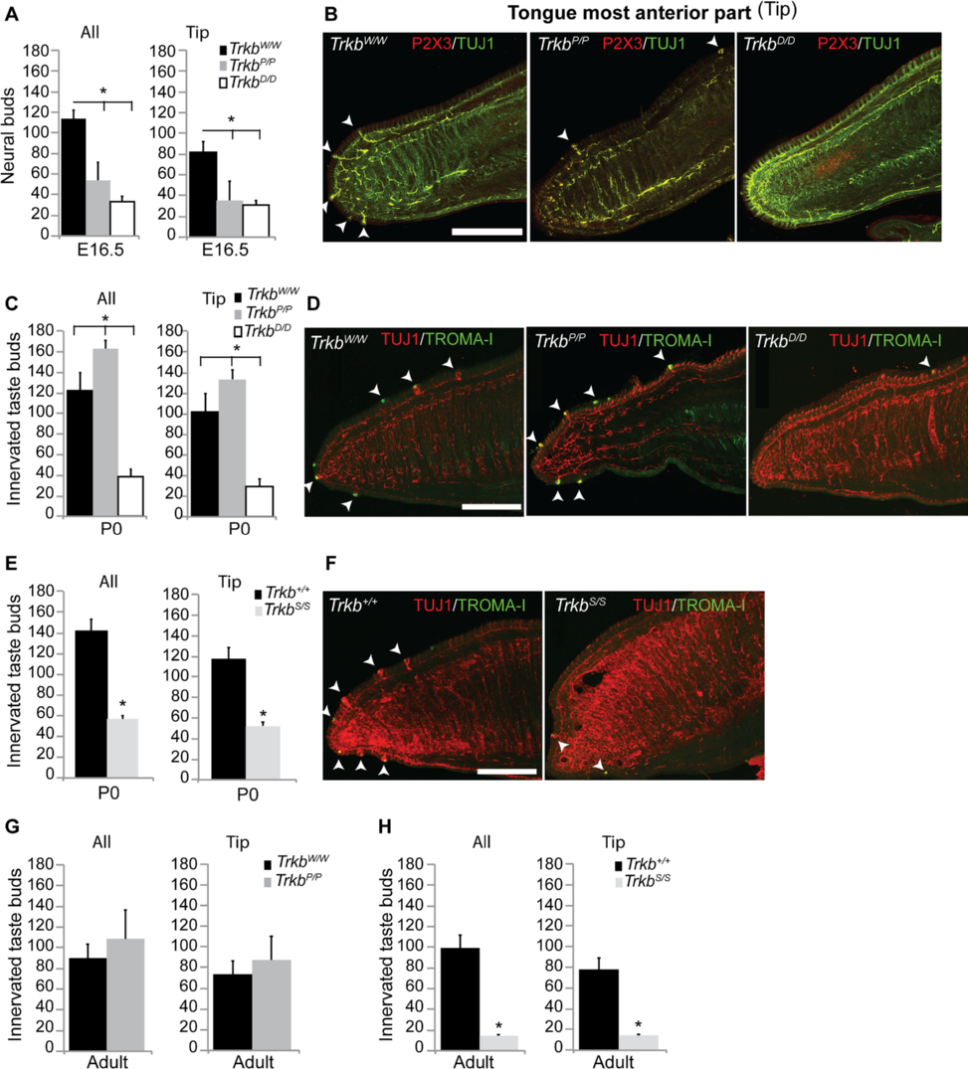
**TrkB PLCγ-site facilitates taste bud innervation during development. (A)** E16.5, all neural buds (*Trkb*^*P/P*^ 54 ± 17.4 vs. *Trkb*^*W/W*^ 113.3 ± 8.3, *P* <0.01; *Trkb*^*D/D*^ 33.3 ± 5, *P* <0.001 vs. *Trkb*^*W/W*^; *P* = 0.119 vs. *Trkb*^*P/P*^); Tip (*Trkb*^*P/P*^ 35.3 ± 18.6 vs. *Trkb*^*W/W*^ 85.3 ± 5.8, *P* <0.01; *Trkb*^*D/D*^ 31.3 ± 4.2, *P* <0.001 vs. *Trkb*^*W/W*^, *P* = 0.734 vs. *Trkb*^*P/P*^), (n = 3) all genotypes. **(B)** E16.5 tongue images (tip) stained with P2X3 and TUJ1 antibodies to visualize geniculate neuron afferents and all neuronal fibres, respectively, as well as neural buds. **(C, E)** P0, all innervated taste buds (*Trkb*^*P/P*^ 163.2 ± 7.8 (n = 5) vs. *Trkb*^*W/W*^ 122.2 ± 17.1 (n = 6), *P* <0.001; *Trkb*^*D/D*^ 38.7 ± 7.57 (n = 3), *P* <0.001 vs. *Trkb*^*W/W*^ and *Trkb*^*P/P*^). Tip (*Trkb*^*P/P*^ (n = 5) 133.6 ± 9.2 vs. *Trkb*^*W/W*^ (n = 6) 101.3 ± 18.2, *P* <0.01; *Trkb*^*D/D*^ 29.3 ± 7.6 (n = 3), *P* <0.001 vs. *Trkb*^*W/W*^ and *Trkb*^*P/P*^). All innervated taste buds (*Trkb*^*S/S*^ 56.3 ± 3.5 vs. *Trkb*^*+/+*^ 142 ± 11.5, *P* <0.001); Tip (*Trkb*^*S/S*^ 52.3 ± 3.2 vs. *Trkb*^*+/+*^ 117 ± 10.7, *P* <0.001), (n = 3) both genotypes. **(D, F)** P0 tongue images (tip) stained with TROMA-I and TUJ1 antibodies to visualize innervations into single taste buds. **(G, H)** Innervated taste buds at 3 months, all (*Trkb*^*P/P*^ 108.3 ± 28.5 vs. *Trkb*^*W/W*^ 90 ± 13.1, *P* = 0.368); Tip (*Trkb*^*P/P*^ 87 ± 23.9 vs. *Trkb*^*W/W*^ 73 ± 13.1, *P* = 0.423). All taste buds (*Trkb*^*S/S*^ 15 ± 1 vs. *Trkb*^*+/+*^ 99.3 ± 12.7, *P* <0.001); Tip (*Trkb*^*S/S*^ 14 ± 1 vs. *Trkb*^*+/+*^ 77.7 ± 11.2, *P* <0.001). (n = 3) all genotypes. *P* statistic from unpaired Student’s *t*-tests, mean ± s.d. Arrowheads indicate neural buds **(B)**, and innervated taste buds **(D, F)** respectively. Scale bars in **B**, **D**, **F**, 400 μm.

Geniculate ganglion lingual afferents innervate and support taste buds in the fungiform papillae that reside in the anterior two thirds of the tongue [16]. This process is known to depend on neurotrophins [17; references therein]. At E14.5, the absence of BDNF in mice reduced the initial innervation of fungiform papillae, and at E16.5 and E18.5, only a few neural buds were evident. Knockouts of *Nt4* do not show this defect at these ages [18]. We therefore examined geniculate lingual innervation in TrkB point mutant mice and respective controls at three stages: E16.5, P0, and adulthood (see below). To identify any regional differences in tongue innervation, we divided the tongue into most anterior (tip) and medial (see Methods) regions, and quantified the number of neural and taste buds in the tip and in the tip + medial regions (all) (Figure 2). In order to distinguish individual neural buds, tongue sections were immunostained for β-III tubulin (TUJ1), a neuronal marker which discerns general neuronal fibre innervation, and for P2X purinoreceptor 3 (P2X3), a specific marker for tongue gustatory afferents [19]. Quantification of lingual neural bud numbers at E16.5 in *Trkb*^*P/P*^, *Trkb*^*D/D*^, and control (*Trkb*^*W/W*^) mice revealed that the amount of all neural buds (tip + medial) in *Trkb*^*P/P*^ and *Trkb*^*D/D*^ animals were similar, and both were significantly reduced compared with controls (Figure 2A,B). Analysis of neural buds on defined regions (tip vs. medial) of the tongue at this age revealed that the tip was significantly affected in the *Trkb*^*P/P*^ mutants as compared with controls (Figure 2A,B), whereas the number of neural buds in the medial region of the tongue, although slightly reduced, was not significantly different compared with controls (*Trkb*^*P/P*^ 18.7 ± 3.1, *Trkb*^*W/W*^ 28 ± 6, *P* = 0.074; *Trkb*^*D/D*^ 2 ± 2, *P* <0.001 compared to controls; n = 3 for all genotypes). These results suggest that, at this stage of development, the TrkB-PLCγ site has a greater influence on the establishment of innervation in the most anterior region of the tongue.

To determine whether the innervation deficit observed in the *Trkb*^*P/P*^ and *Trkb*^*D/D*^ point mutants is restored at a later stage, we examined fungiform taste bud innervation at birth (P0). While *Trkb*^*D/D*^ animals showed an amount of innervated taste buds comparable to that seen at E16.5, likely reflecting the loss of the geniculate neurons in these mice (Figure 2C), in contrast, the *Trkb*^*P/P*^ animals showed an increase in innervated taste buds that exceeded the number in control animals at this age (Figure 2C), both in the tip (Figure 2C,D), and medial regions of the tongue (*Trkb*^*P/P*^ 29.9 ± 3.6 vs. *Trkb*^*W/W*^ 20.8 ± 4.8, *P* <0.01). Analysis of taste bud innervation in *Trkb*^*S/S*^ mutants, as expected from the loss of geniculate neurons in these animals, revealed significantly fewer innervated taste buds both in the anterior two thirds of the tongue and in the tip of the tongue when compared with control littermates (Figure 2E). Based on previous work that has established the requirement of BDNF for innervation of the fungiform papillae by E14.5 of development [17; references therein], we conclude that absence of the PLCγ docking site in TrkB delays the timing of tongue epithelium innervation by geniculate neurons; however, an intact SHC-site on the TrkB receptor is sufficient to rescue taste bud innervation by birth.

### TrkB-PLCγ site is not required for the maintenance of adult fungiform taste bud innervation by geniculate neurons

It has previously been demonstrated that taste buds require innervations for their maintenance and that loss of *Bdnf* induces significant loss of fungiform taste buds [17,20,21]. In adult animals, the taste system is fully developed, contains no or very few un-innervated taste buds, and is capable of detecting the full complement of tastes [22]. In order to determine the involvement of TrkB docking sites in a developed gustatory system, we first analyzed the number of innervated taste buds present in adult animals carrying a point mutation either at the SHC- or the PLCγ-docking site. Unlike the P0 stage, the analysis revealed similar numbers of innervated fungiform taste buds in the tongues of *Trkb*^*P/P*^ mice compared with *Trkb*^*W/W*^ control animals (Figure 2G). Similar numbers of innervated fungiform taste buds were also found in the most anterior part of the tongue (tip) between these two genotypes (Figure 2G), as well as in the medial area (*Trkb*^*P/P*^ 21.3 ± 6.4 vs. *Trkb*^*W/W*^ 20.3 ± 58, *P* = 0.85). On the other hand, the *Trkb*^*S/S*^ mutant mice showed significantly reduced fungiform taste bud innervation compared with control littermates (Figure 2H). Similar results were obtained for innervated taste buds in the tip area of the tongue (Figure 2H). The decrease was comparable to that seen in the *Trkb*^*D/D*^ mice at birth. These results suggest that the TrkB-PLCγ site is not required to maintain the innervation of adult fungiform taste buds, whereas the TrkB-SHC site is crucial for the survival of TrkB-dependent geniculate neurons, and the maintenance of their innervation to fungiform taste buds.

### Fungiform taste bud morphology at birth

Taste buds require two to seven geniculate ganglion axons in order to survive [23]. Moreover, the amount of innervation determines the morphology of taste buds [24]. We therefore examined the morphology and measured the volume of fungiform taste buds on the anterior two thirds of tongues of the different TrkB mutant strains at birth.

**Figure 3:**
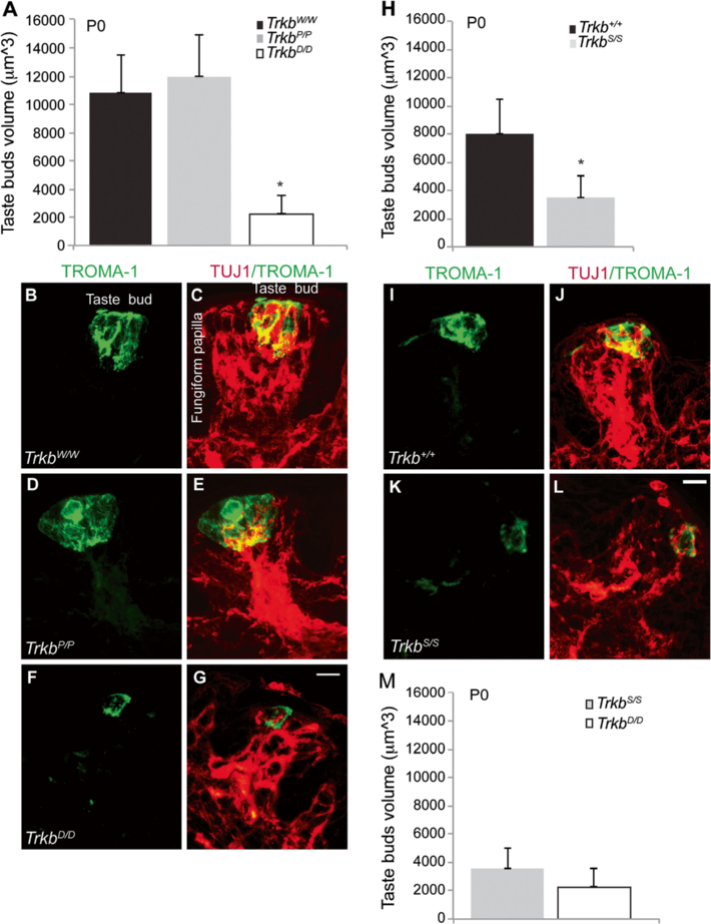
**Morphological analysis of innervated fungiform taste buds at birth. (A)** Volume of taste buds was measured in point mutants and respective controls at birth (P0) as a readout for morphology (*Trkb*^*P/P*^ 11,999 ± 2,868 μm^3^ vs. *Trkb*^*W/W*^ 10,784 ± 2,743 μm^3^, *P* = 0.904), n = 3 for each genotype (taste buds analysed: *Trkb*^*P/P*^ n = 21; *Trkb*^*W/W*^ n = 28); (*Trkb*^*D/D*^ 2,296 ± 1,254 μm^3^), n = 3 (n = 14 taste buds), *P* <0.001 vs. *Trkb*^*W/W*^ and *Trkb*^*P/P*^. **(B–G)** Representative images of tongue sections double immunostained with TROMA-I and TUJ1 antibodies to visualize taste buds **(B, D, F)** and innervated taste buds in fungiform papillae **(C, E, G)** for *Trkb*^*W/W*^ control, *Trkb*^*P/P*^, and *Trkb*^*D/D*^ mutants. **(H)** Taste bud morphology of *Trkb*^*S/S*^ mutants and respective controls. Volume (*Trkb*^*S/S*^ 3,537 ± 1,504 μm^3^ vs. *Trkb*^*+/+*^ 8,006 ± 2,497 μm^3^, *P* <0.001), n = 3 each genotype (taste buds: *Trkb*^*+/+*^; n = 24; *Trkb*^*S/S*^ n = 14). **(I–L)** TROMA-1 immunostaining highlights taste buds in *Trkb*^*+/+*^ and *Trkb*^*S/S*^**(I, K)**. Innervated taste buds within the fungiform papillae are revealed by double immunostaining of TROMA-1 and TUJ1 antibodies **(J, L)**. **(M)** Comparison between *Trkb*^*S/S*^ and *Trkb*^*D/D*^ mutant taste bud morphology volume (*Trkb*^*S/S*^ vs. *Trkb*^*D/D*^, *P* = 0.403), revealing similar reduction in fungiform taste bud size of the two genotypes. *P* statistic from unpaired Student’s *t*-test; mean ± s.d. Scale bars, **(G, L)** 10 μm.

*Trkb*^*P/P*^ taste bud morphology at birth was not significantly different compared with control taste buds (Figure 3A–E). However, *Trkb*^*D/D*^ taste buds were morphologically different and significantly smaller than control and *Trkb*^*P/P*^ taste buds (Figure 3A,D–G). This likely reflects the lack of innervation resulting from the loss of neurons in the geniculate ganglia during development, supporting the fact that taste buds need innervation to properly develop and mature.

A reduction in fungiform taste bud size similar to that found in the *Trkb*^*D/D*^ mutants at birth was observed in *Trkb*^*S/S*^ mutant tongues (Figure 3H–M). The lack of a significant difference at this age between *Trkb*^*S/S*^ and *Trkb*^*D/D*^ mutants likely reflects the TrkB-dependent loss of taste neurons in these two genotypes, causing a lower amount of geniculate fibres being available to innervate taste buds on the tongue, and consequently a decrease in the size of the taste buds in these animals at this age.

### Morphology of remaining fungiform taste buds recovers in adult *Trkb*^*S/S*^ mice

**Figure 4:**
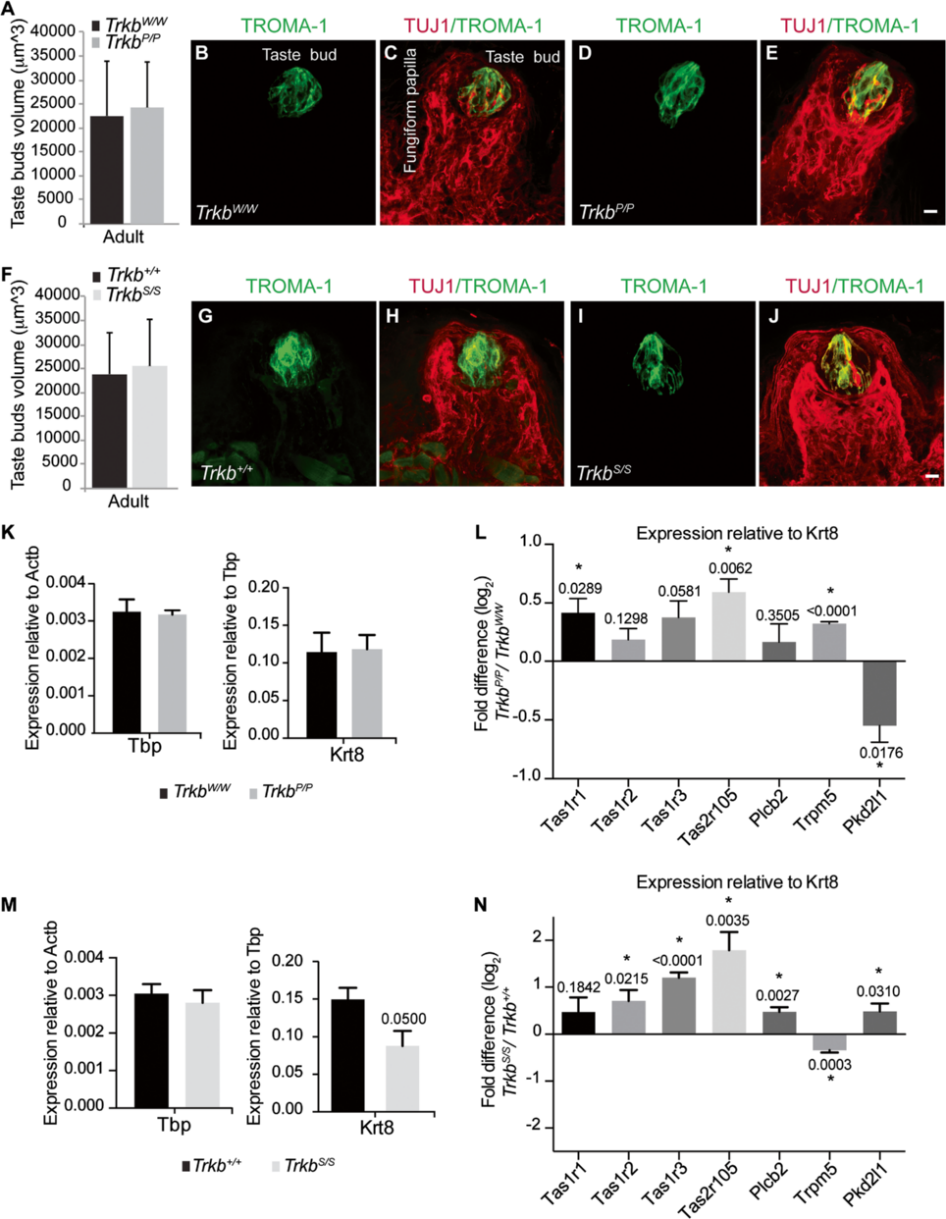
**Morphology of adult fungiform taste bud and taste receptor analysis. (A)** Taste bud volume, 3 to 4 months old mice (*Trkb*^*P/P*^ 24,290 ± 9,377 μm^3^ vs. *Trkb*^*W/W*^ 22,539 ± 11,319 μm^3^, *P* = 0.427); (n = 3) all genotypes (taste buds: *Trkb*^*P/P*^ n = 50; *Trkb*^*W/W*^ n = 39). Values are mean ± s.d., *P* statistic from unpaired Student’s *t*-tests. **(B–E)** Images of adult innervated taste buds within fungiform papillae highlighted by double immunostaining with TROMA-1 and TUJ1 antibodies. **(F)** Volume of taste buds in adult (3 months old) *Trkb*^*S/S*^ and controls (*Trkb*^*S/S*^ 25,526 ± 9,746 μm^3^ vs. *Trkb*^*+/+*^ 23,849 ± 8,539 μm^3^, *P* = 0.533); (n = 3) each genotype (taste buds for *Trkb*^*S/S*^ n = 17; *Trkb*^*+/+*^ n = 33). Values are mean ± s.d., *P* statistic from unpaired Student’s *t*-tests. **(G–J)** Images of *Trkb*^*+/+*^ and *Trkb*^*S/S*^ adult innervated taste buds within fungiform papillae highlighted by double immunostaining with TROMA-1 and TUJ1 antibodies. Scale bar, **(E, J)** 10 μm. **(K–N)** qRT-PCR analysis. Expression analysis of TATA-binding protein (Tbp) relative to beta actin (Actb) (left), and Cytokeratin-8 (Krt8) relative to Tbp (right) for *Trkb*^*P/P*^ mice (n = 3) and respective controls (*Trkb*^*W/W*^, n = 3) **(K)**. **(L)** Fold difference of gene expression of various taste receptors in tongue tissues between *Trkb*^*W/W*^ controls and *Trkb*^*P/P*^ mutants (n = 3 per genotype, values are mean ± s.e.m., *P* statistic from unpaired Student’s *t*-tests). **(M)** Expression analysis of Tbp relative to Actb (left), and Krt8 relative to Tbp (right) for adult *Trkb*^*S/S*^ mice (n = 4) and respective controls (*Trkb*^*+/+*^, n = 4). **(N)** Fold difference of gene expression of various taste receptors in tongue tissues between *Trkb*^*+/+*^ controls and *Trkb*^*S/S*^ mutants (n = 4 per genotype, values are mean ± s.e.m., *P* statistic from unpaired Student’s *t*-tests).

The development of the gustatory system in rodents is achieved after birth; however, taste buds grow to their full adult size in the first month of life. This process suggests a clear correlation between the number of innervating geniculate ganglion cells and taste bud size [24]. We therefore measured the volume of fully developed taste buds in adult tongues of the *Trkb*^*P/P*^ and *Trkb*^*S/S*^ point mutants and their respective controls. As seen at P0, no significant morphological differences were found between fungiform taste buds of *Trkb*^*P/P*^ mutant and *Trkb*^*W/W*^ control mice (Figure 4A–E). Despite *Trkb*^*S/S*^ mutants exhibiting significantly smaller fungiform taste buds at birth, the difference was not apparent at 3 to 4 months of age (Figure 4F–J). Therefore, the remaining TrkB-independent geniculate neurons are able to innervate and control the morphological development and maturation of the residual fungiform taste buds in *Trkb*^*S/S*^ mutant tongues after birth. *Trkb*^*D/D*^ animals die between 2 to 3 weeks of age and therefore could not be included in the analysis [15].

### Taste receptors (TRs) for taste detection in TrkB point mutants

Finally, taste buds are composed of taste receptor cells (TRCs), which are considered to be the anatomical substrate for taste detection. These cells transduce gustatory information upon activation of specific TRs located on their surface and convey taste information to gustatory afferent fibres [25,26]. There are distinct classes of both TRCs and TRs that ultimately allow different tastes to be distinguished. In particular, Type II TRCs express sweet, bitter, and umami classes of TRs. Sweet and umami tastes are sensed by heterodimeric G protein-coupled receptors (GPCRs) of similar subunits, arranged in different combinations (taste receptor, type 1, member 1 (T1R1)/T1R3 for umami, and T1R2/T1R3 for sweet), whilst bitter tastes activate the GPCR T2R taste receptor class. In contrast, Type III TRCs lack expression of receptors for sweet, bitter, and umami tastes, but express sour TRs such as transient receptor potential (TRP) ion channel and polycystic kidney disease 2-like protein 1 (PKD2L1) [26].

In order to determine whether mutation of the SHC or PLCγ docking sites in TrkB would affect any particular TR combination and eventually functionality of the mature taste buds, we examined the lingual expression of different TRs known to transduce different taste modalities in the different point mutant mice. In particular, RNA was extracted from the anterior two thirds of tongues of 2- to 3-month-old mice and subjected to quantitative RT-PCR analysis for GPCRs T1R1 (Tas1r1), T1R2 (Tas1r2), T1R3 (Tas1r3), and T2R5 (Tas2r105), as well as PLC-β2 and TRPM5, which are downstream effector and transduction channels, respectively, of the sweet, umami, and bitter taste pathways. In addition the expression of PKD2L1, a putative sour TR, was quantified.

Figure 4K,M demonstrates that the mRNA of the housekeeping gene TATA-binding protein (Tbp) was present at similar levels in all point mutant and respective control tongue samples, denoting isolation of intact RNA. To analyse the expression levels of each TR in the taste buds we used the taste bud marker, Cytokeratin-8, as a reference gene. In the mouse oral cavity this gene is exclusively expressed in taste buds [27]. Similar levels of Cytokeratin-8 were found between *Trkb*^*P/P*^ mutant mice and their respective controls (Figure 4L), however, the umami TR subunit (Tas1r1/T1R1), but not the sweet subunit (Tas1r2/T1R2) was significantly upregulated in *Trkb*^*P/P*^ animals, as was the bitter TR Tas2r105/T2R5 (a candidate cycloheximide receptor) (Figure 4L). Together with an observed significant increase in the TRPM5 channel expression, these results suggest that deactivation of the PLCγ − activated pathway downstream of the TrkB may potentiate the response to umami and bitter tastes, but down-regulate the response to sour tastes, since the expression of the TRP-like channel PKD2L1 was found to be significantly decreased in the TrkB-PLCγ mutants (Figure 4L). A similar analysis of lingual gene expression in *Trkb*^*S/S*^ point mutants revealed decreased Cytokeratin-8 levels in these animals compared with controls, corroborating the observation that very few taste buds remain in these mutants (Figure 4M). Analysis of the different TRs in these mutants showed a significant up-regulation of the sweet TR subunit (Tas1r2/T1R2), and the common subunit Tas1r3/T1R3, as well as an up-regulation of the bitter receptor Tas2r105/T2R5 and the effector PLC-β2, a component of the canonical PLC-signalling cascade (Figure 4N). In contrast, the TRPM5 channel was significantly down-regulated in the *Trkb*^*S/S*^ mutants suggesting an alteration in the transduction of sweet, bitter, and umami tastes (Figure 4N). However, the expression of PKD2L1, involved in sour taste quality, was significantly up-regulated in *Trkb*^*S/S*^ point mutants compared to controls (Figure 4N). These data suggest that in *Trkb*^*S/S*^ point mutants either remaining taste buds alter their TR composition to accentuate taste qualities, or these TrkB-independent taste buds facilitate mainly sour taste transduction.

## Conclusions

Understanding and advancing the biology of the taste system is undoubtedly important as this system, together with the olfactory system, is fundamental for an organism to relate to the environment. Herein, we have genetically dissected pathways activated downstream of the TrkB receptor that are required for specific aspects of the taste system regulated by the two neurotrophins NT-4 and BDNF. In addition, we have reported that both TrkB docking-sites modulate the expression of specific TRs. These results advance our knowledge in the biology of the taste system in general, and in particular underpin the mechanisms underlying neurotrophin-signalling control of the gustatory system development in rodents.

## Methods

### Ethics statement

All animal procedures conformed to the UK legislation Animals (Scientific procedures) Act 1986 and to the University of Edinburgh Ethical Review Committee (ERC) policy as well as the University of Oxford ERC policy.

### Mouse strains

TrkB signalling point mutant lines, *Trkb*^*SHC*^, *Trkb*^*PLC*^, and *Trkb*^*D*^, and the control line, *Trkb*^*W*^, which is a direct control for the knock-in strategy used to generate the *Trkb*^*PLC*^ and the *Trkb*^*D*^ strains, have been previously described [13-15]. The animals used in these experiments had a mixed genetic background (C57B6/129). All experiments were carried out by an experimenter blind to genotype.

### Histology and geniculate neuron counts

Embryos were collected at stages E12.5 and E14.5 using timed matings. Their heads were dissected and fixed in ice-cold 4% paraformaldehyde (PFA) and left at 4°C overnight. Tissues were then dehydrated by incubation in a saline solution of increasing alcohol concentrations, cleared in xylene and embedded in paraffin. P4 pups were transcardially perfused with 4% PFA. Their heads were post-fixed in the same solution at 4°C for 3 days, decalcified in 5% formic acid in PBS, dehydrated, and embedded in paraffin. Serial transversal sections of either 7 μm (E12.5 and E14.5 tissues) or 8 μm (P4 tissues) were mounted on SuperFrost Plus Slides (VWR) and stained using hematoxylin and eosin. Bright-field images of the whole geniculate ganglion were taken from each section with an Axio Scope (Carl Zeiss) microscope using 40×/0.75 NA plan-apochromat objective, and analysed using ImageJ software (http://rsb.info.nih.gov/ij/). Total numbers of geniculate ganglion neurons per animal were estimated using previously described methods [5,6,10]. Briefly, the total volume of the geniculate ganglion of each animal was estimated by calculating the volume of the ganglion in every fourth section as follows: [ganglion area × section thickness], and then summing the obtained values over all sections analysed. Neuron profiles were identified by virtue of the hematoxylin staining and nuclei counted in every fourth section. The neuronal density per section was then calculated as follows: [number of neurons/(ganglion area × section thickness)]. The mean of the neuronal densities of all counted sections was then multiplied by the volume of the total ganglion to calculate the total number of neuronal profiles per ganglion. Values for each ganglion were corrected for split nuclei by a correction factor, according to the following formula: N = n × [T/(T × D)], where ‘N’ is the estimated total number of neurons, ‘n’ is the number of nuclear profiles, ‘T’ is the measured section thickness, and ‘D’ is the average diameter of the nuclei. The correction formula is based on previous literature [5,6,10].

### Tongue immunostaining

Embryos at E16.5, pups at birth (P0), and adult mice (3 months old) (n = 3 in each case) were perfused with ice-cold 4% PFA. Dissected tongues were post-fixed in 4% PFA at 4°C overnight, then washed in PBS and placed in 30% sucrose at 4°C overnight. Tissues were then embedded in OCT mounting medium (VWR) and serially sectioned in the sagittal plane at 50 μm. Sections were washed 4 × 15 minutes in 0.1 M phosphate buffer (pH 7.4) (PB), followed by an antigen retrieval procedure for adult sections. In this case, each slide was subjected to 10 minutes of 200 μL of proteinase K in 0.1 M PB at 20 μg/mL. This was followed by a wash in 0.1 M PB 2 × 10 minutes and washed in quenching solution (0.05% NaN_3_, 0.1 M glycine, 0.1 M NH_4_Cl, 50 mM Tris) for 30 minutes at room temperature. Sections were then washed 2 × 15 minutes in 0.1 M PB and blocked overnight at 4°C in blocking solution (3% NGS, 0.5% Triton-X 100, 0.1% NaN_3_ in 0.1 M PB) and the next day they were incubated in primary antibody solution (200 μL per slide). Serial tongue sections spaced 100 μm, were co-immunostained using the following antibodies: anti-β III tubulin (TUJ1, 1:300, R&D Systems) and either anti-P2X3 (P2X3, 1:500, Millipore) for E16.5 tissues, or anti-cytokeratin 8 (TROMA-1, 1:200, Developmental Studies Hybridoma bank) for P0 samples. Slides were covered with parafilm and left at 4°C for 5 days. Following primary antibody staining, the sections were washed 4 × 15 minutes in 0.1 M PB and incubated in secondary antibody overnight at 4°C. The conjugated secondary antibodies used were Alexa-488 anti-mouse (1:1,000, Invitrogen) and Alexa-555 anti-rabbit (1:1,000, Invitrogen) for E16.5 samples; or FITC anti-rat (1:1,000, Abcam) and Alexa-555 anti-mouse (1:1,000, Invitrogen) for P0 samples. Sections were washed with 0.1 M PB, and mounted in Vectashield. Tissues were imaged with a Zeiss LSM710 Meta confocal microscope. For E16.5 and P0 tissues, a plan-apochromat 20×/0.8 M27 objective was used, and the whole section was imaged with a 3 × 7 tile scan and 4-μm Z-steps. This imaged the whole section of the tongue, so that the innervation into the neural/taste buds could be analyzed. Adult tissues were imaged with a plan-apochromat 10×/0.45 objective and 8-μm Z-steps through a 4 × 6 tile scan.

### Analysis of tongue innervation

To analyse innervated fungiform taste buds in specific regions of the tongue, immunolabelled tongues at different stages were subdivided into two different regions, the tip and the middle. At E16.5, the tip of the tongue was defined as the anterior-most 1,250 μm of the tongue, while the middle as the part following the tip of the tongue from 1,250 to 2,500 μm. At birth, the tip was considered as the anterior-most 1,750 μm of the tongue, the middle following the tip of the tongue from 1,750 to 3,500 μm. In adult tongues, the tip was considered as the anterior-most 4,000 μm of the tongue, and the middle area of the tongue from 4,000 to 8,000 μm. ImageJ software was used to score for the presence of innervated taste buds.

### Taste bud morphology

Tongue sections used for the innervation analysis were also examined for taste bud morphology. Innervated taste buds visualized by double positive for Tuj1 and TROMA-1 staining were randomly selected from the tip and the middle regions of the tongue. High magnification images were taken with a Zeiss LSM710 Meta confocal microscope using a plan-apochromat 63×/1.40 Oil DIC M27 objective. Images were taken every 1 μm scanning for both 555 nm and 488 nm wavelengths. They were then analysed with ImageJ software; using the function “Z Project”, the images were amalgamated into maximum intensity, and the widest and highest points measured using the “straight” tool. These values were then multiplied by the depth of each taste bud, thus evaluating the volume of a given taste bud. Identical analysis was performed on both P0 and adult taste buds.

### qRT-PCR analysis

Male mice at 2 to 3 months of age were culled by cervical dislocation. Their tongues were removed and the anterior two thirds were quickly dissected and frozen. Total RNA was isolated using Trizol (Invitrogen). cDNA synthesis was performed using Superscript VILO cDNA synthesis kit (Invitrogen) as per the manufacturer’s instructions. Quantitative PCR was performed using UPL assays (Roche) and the CFX384 Touch™ Real-Time PCR Detection System (Bio-Rad). Gene specific primers were designed using the Universal ProbeLibrary System Assay Design Centre (Roche) and their sequences were as follows: Tas1r1/T1R1 (Forward (For) 5’-actgctgcttcgagtgcat-3’ , Reverse (Rev) 5-acaaggctggcaggtgtg-3’), Tas1r2/T1R2 (For 5’-gtactcggccgtctacgc-3’ , Rev 5’-catgccagatctccctgagt3’), Tas1r3/T1R3 (For 5’-aggccactctcaaccagaga-3’ , Rev 5’-gaacaaaccaaggggtgaga-3’), Tas2r105/T2R5 (For 5’-ctgcatgtttcttgttaattatgtca-3’ , Rev 5’-tcctgaaacaccagactgcat-3’), Plcb2 (For 5’-cagtggaccgcattgatgt-3’ , Rev 5’-acaggaactgcccagagatg3’), Trpm5 (For 5’-aaacggaggagggacagc-3’ , Rev 5’-ccgatgtatttggcaatcaa-3’), and Pkd2l1 (For 5’-ctggacctggtggtcatctt-3’ , Rev 5’-gggttcggaatatgtggaaa-3’). The threshold cycle of Ct value was calculated using the CFX Manager™ software (Bio-Rad). Ct values (threshold cycle) of each gene were normalized to that of Tbp (ΔCt), Actb, or Cytokeratin-8 in the same sample, as indicated in Figure 4. Data are presented as fold difference between control and mutants using the ΔΔCt method.

### Data analysis

Sample sizes were based on pilot experiments. Statistical analysis was performed using StatView and GraphPad Prism software. Mean values were compared using two-tailed Student’s *t*-tests or two-way ANOVA followed by Fisher’s Protected Least Significant Difference post-hoc tests. For qRT-PCR analysis, unpaired Student’s *t*-tests were used. Differences were considered significant when *P* <0.05.

## Abbreviations

BDNF: Brain-derived neurotrophic factor; E: Embryonic day; GPCR: G protein-coupled receptor; NT-4: Neurotrophin-4; P: Postnatal day; P2X3: P2X purinoreceptor 3; PKD2L1: Polycystic kidney disease 2-like protein 1; PLC-β2: Phospholipase Cβ2; PLCγ: Phospholipase Cγ; SHC: Sh2-domain containing protein; T1R1: Taste receptor, type 1, member 1; Tbp: TATA binding protein; TR: Taste receptor; TRC: Taste receptor cell; TrkB: Tropomyosin-receptor kinase B; Trkb^+/+^: Genotype denoting homozygous wild-type sequences encoding for TrkB; Trkb^S/S^: Genotype denoting a homozygous point mutation in the genomic sequence encoding for Y515 (the SHC docking site) of TrkB; Trkb^W/W^: Genotype denoting a homozygous insertion of wild-type cDNA sequence encoding for Y816 of TrkB; Trkb^P/P^: Genotype denoting a homozygous point mutation in inserted cDNA sequence encoding for Y816 (the PLCγ docking site) of TrkB; Trkb^D/D^: Genotype denoting homozygous point mutations in inserted cDNA sequence encoding for Y515 (the SHC docking site) and for Y816 (the PLCγ docking site) of TrkB; TROMA-I: Antibody recognizing Cytokeratin-8; TRP: Transient receptor potential; TRPM5: Transient receptor potential cation channel, subfamily M, member 5; TUJ1: Antibody recognizing neuron-specific class III beta-tubulin; Y: Tyrosine.

## Competing interests

The authors declare that they have no competing interests.

## Authors’ contributions

JK performed most of the experiments. JMH managed the genetically modified strains. CV performed the RT-PCR data. LM contributed to the experimental plans, provided theoretical input and supervision, and wrote the manuscript. All authors read and approved the final manuscript.
